# Causes and risk factors for death in infants with congenital chylothorax

**DOI:** 10.3389/fped.2025.1699515

**Published:** 2025-11-14

**Authors:** Yuichi Kubo, Satoshi Ibara, Takuya Tokuhisa, Masaya Kibe, Kazuyoshi Sueyoshi, Masato Kamitomo, Motoi Kato

**Affiliations:** 1 Department of Neonatology, Shonan Fujisawa Tokushukai Hospital, Kanagawa, Japan; 2 Department of Neonatology, Kagoshima City Hospital, Kagoshima, Japan; 3 Department of Neonatology, Imakiire General Hospital, Kagoshima, Japan; 4 Department of Pathology, Kagoshima City Hospital, Kagoshima, Japan; 5 Department of Obstetrics, Kagoshima City Hospital, Kagoshima, Japan; 6 Department of Plastic Surgery, Kagoshima University Hospital, Kagoshima, Japan

**Keywords:** congenital chylothorax, cause of death, lymphangiectasia, infant mortality, risk factors, chromosome abnormalities

## Abstract

**Aim:**

To identify mortality risk factors in infants with congenital chylothorax (CC) and analyze the causes of death, including pathological examination.

**Methods:**

For this single-center retrospective study, we included 27 patients with CC. We divided them into the ALIVE and the DEAD groups, with the DEAD group subdivided into the Early-DEAD group and the Late-DEAD group to compare patient characteristics and risk factors using robust statistical methods. Causes of death were reviewed, including pathological findings from autopsies.

**Results:**

The mortality rate was 44% (12 of 27 infants, with 15 survivors). Univariate analysis showed associations between mortality and chromosomal abnormalities and fetal ascites. A longer duration of hydrothorax *in utero* was specifically associated with early neonatal death. Multivariate regression analysis identified fetal ascites as the strongest independent predictor of mortality. Pathological examination of six autopsied fatal cases revealed lymphangiectasia, including systemic lymphangiectasia in all five whole-body autopsies.

**Conclusion:**

Poor prognosis in infants with CC is strongly associated with fetal ascites, which we hypothesize serves as a key clinical marker for underlying Generalized Lymphatic Dysplasia (GLD). This systemic lymphatic disorder, identified pathologically as lymphangiectasia in fatal cases, may be the primary driver of mortality. Chromosomal abnormalities appear to be a significant predisposing factor for this severe phenotype.

## 1 Introduction

Congenital chylothorax (CC), defined as hydrothorax caused by leakage of lymphatic fluid from a thoracic duct, is one of the major causes of fetal hydrothorax. However, it is rare, with a prevalence rate of approximately 4.6–17/100,000 births (1, 2). Previous studies on the course and prognosis of CC (1–11) have reported a mortality rate of up to 44% (1–3, 5, 7, 8, 10, 11). Although CC is considered a critical disease, few studies have investigated the causes and risk factors of death.

Therefore, we aimed to identify risk factors associated with mortality in infants with CC by comparing patients who died during hospitalization with those who survived. Furthermore, we examined the causes of death, including pathological findings from patients who underwent autopsy.

## 2 Materials and methods

### 2.1 Data collection

This monocentric case-control study was performed at our neonatal intensive care unit in Kagoshima City Hospital, the tertiary care center of Kagoshima Prefecture in Japan. We searched patient admission records over a 15-year period, from January 1, 2005, to December 31, 2019, and enrolled all cases with CC. Patients were diagnosed with CC if they showed pleural effusion *in utero* or on the day of birth, and if the total cell count of the pleural tap was over 1,000 /mm^3^ with lymphocytes accounting for more than 80% of the cells (12). Imaging studies specific to the lymphatic system, such as contrast lymphangiography or magnetic resonance lymphangiography, were not performed as they were not standard practice for neonates at our institution during the study period.

Data collection included congenital anomalies, chromosomal abnormalities, sex (male or not), gestational age at birth, birth weight, Apgar score, antenatal steroid use, gestational age at the onset of hydrothorax *in utero* (CC_start_week), the duration from detection of chylothorax until birth *in utero* (Fetal_CC_Days), and whether thoracocentesis or thoraco-amniotic shunt placement was performed, according to past studies (3–5, 8). We excluded patients with complex congenital heart disease due to the risk of death from heart failure unrelated to CC.

### 2.2 Patient assignment

We divided eligible infants into two groups: the ALIVE and the DEAD groups. Patients in the ALIVE group were discharged alive, while those in the DEAD group died during hospitalization. Furthermore, we subdivided the DEAD group into the Early-DEAD [patients who died within 7 days of life (DOL)] and the Late-DEAD (patients who died after 7 DOL) groups. This was done to evaluate risk factors for death depending on the timing of death.

### 2.3 Statistical analysis

Continuous variables are presented as means with standard deviations (SDs) or medians with ranges. Categorical variables are presented as frequencies and percentages. In the univariate analysis, categorical variables were evaluated using Fisher's exact test depending on the number of data points, and continuous variables were evaluated using the Mann–Whitney U test owing to non-normal data distribution. All tests were one-sided to confirm that the group with the lower median was truly lower.

Additionally, we calculated the *post hoc* power of the test between the ALIVE and the DEAD groups because our study was retrospective and we could not determine the sample size in advance. Power analysis was conducted using G*Power version 3.1.9.7 (13), with an effect size set at 0.5 and significance level at 0.05.

To identify risk factors for mortality, logistic regression models were constructed. However, due to the small sample size and the issue of ‘complete separation'—where a predictor (i.e., chromosomal abnormalities) perfectly predicts the outcome for a subgroup—standard maximum likelihood logistic regression was deemed inappropriate for providing stable estimates. Therefore, we adopted more robust methods. For the univariate analysis of categorical predictors, Fisher's exact test was used to calculate odds ratios (ORs), 95% confidence intervals (95% CIs), and *p*-values. For the multivariate analysis, we employed Firth's penalized likelihood logistic regression to assess the independent effect of each predictor. Predictor variables included gestational week (GW), male sex, and Apgar score at 5 min (Apgar_5), according to previous studies (3–9). Furthermore, items showing significant differences between the ALIVE and the DEAD, the Early-DEAD, or the Late-DEAD groups were also used as predictor variables. In addition, we used the estimated model to predict the probability of mortality, classifying it as 1 when the probability was greater than 0.5 and as 0 when it was less than 0.5. The performance of the prediction was evaluated using a confusion matrix. The dataset used for model estimation and validation was identical because CC is a rare disease and our sample size was limited, making it impractical to prepare a separate dataset.

All other statistical analyses were performed using R statistical software version 4.0.2 (The R Foundation for Statistical Computing) (14). Statistical significance was set at a one-sided *P*-value of less than 0.05.

### 2.4 Causes of death and pathological analysis

The cause of death was defined as the clinical diagnosis recorded in each patient's medical record. We also evaluated whether CC had resolved before death to determine whether CC itself could be the cause of death. In addition, we reviewed pathological findings for all patients who underwent autopsy. Samples from autopsy were re-stained with D2–40 to identify the characteristics of the lymphatic duct (15–17).

### 2.5 Ethical considerations

This study was conducted in accordance with the provisions of the Declaration of Helsinki. The study protocol was approved by the Institutional Review Board of Kagoshima City Hospital (Approval No. 2020–23). In accordance with the guidelines for observational studies in Japan, consent was obtained from the parents or guardians of all participants using an opt-out format, where the research details were presented on the hospital's website, and families were given the opportunity to decline participation.

## 3 Results

### 3.1 Patient characteristics

A total of 27 patients were diagnosed with CC in our hospital during the study period. No infant with complex congenital heart disease was included; hence, all 27 patients were analyzed. All mothers in the study cohort had received regular prenatal check-ups, which allowed us to accurately evaluate the timing of the onset of pleural effusion *in utero*.

Among the study cohort, 12 patients (44%) died during hospitalization, while the remaining 15 were discharged alive. Five patients had chromosomal abnormalities, and all of them died. The chromosomal abnormalities included three patients with trisomy 21, one with 7p interstitial deletion, and one mosaicism with a supernumerary marker chromosome (mos 47,XX,+mar/46,XX). In addition to chromosomal abnormalities, one patient had a coloboma in the right eye (ALIVE), one had multiple intestinal perforations (DEAD), one had a vein of Galen malformation (ALIVE), one had myotonic dystrophy (ALIVE), and one had clinically diagnosed Noonan syndrome (DEAD).

### 3.2 Comparison between the ALIVE and the DEAD groups

The power of the test exceeded 0.8 only for chromosomal abnormalities. The rates of chromosomal abnormality (0% vs. 41.7%, respectively) and fetal ascites (20% vs. 83.3%, respectively) were significantly lower in the ALIVE group compared with the DEAD group (Table 1). Other variables, such as GW and birth weight, did not show statistically significant differences.

**Table 1:** Patient characteristics.

	ALIVE (*n* = 15: 56%)	DEAD (*n* = 12: 44%)	*p*	power	Early-DEAD (*n* = 4)	*p*	Late-DEAD (*n* = 8)	*p*
Chromosomal abnormality	0 (0%)	5 (41.7%)	<0.05	0.81	2 (50%)	<0.05	3 (37.5%)	<0.05
GW	34 (32.5–34.5)	33 (32–34)	0.15	0.23	33 (31.75–34.0)	0.16	33 (32.75–33.5)	0.24
Birth weight	2,520 (2,080–2,912)	2,098 (1,616–2,878)	0.2	0.23	2,614 (2,266–2,804)	0.56	1,699 (1,484–2,878)	0.87
Male	5 (33.3%)	5 (41.7%)	0.48	0.04	1 (25%)	0.63	4 (50%)	0.37
Apgar_1	3 (2.5–4.5)	1.5 (1.0–4.25)	0.12	0.23	2.5 (1.75–3.25)	0.18	1.0 (1.0–5.0)	0.18
Apgar_5	6 (5–7)	5.5 (4–6)	0.15	0.23	5.0 (3.75–6.0)	0.13	5.5 (4–6.5)	0.28
UA_pH	7.295 (7.274–7.329)	7.266 (7.19–7.303)	0.06	0.23	7.288 (7.273–7.303)	0.33	7.228 (7.28–7.299)	<0.05
Fetal_CC_Days	12.0 (2.5–19.5)	21.0 (14.0–30.0)	0.11	0.23	48.0 (32.0–65.5)	<0.05	14.5 (10.5–21.0)	0.4
CC_start_week	32 (29.5–33.0)	29.5 (26.0–31.5)	0.12	0.23	25.0 (23.75–26.75)	<0.05	31.0 (29.75–33.0)	0.45
Fetal Ascites	3 (20%)	10 (83.3%)	<0.05	0.23	3 (75%)	0.07	7 (87.5%)	<0.05
Hydrops	13 (86.7%)	10 (83.3%)	0.61	0.02	4 (100%)	1	10 (83.3%)	0.43
Antenatal_steroid	5 (33.3%)	5 (41.7%)	0.48	0.04	1 (25%)	0.63	5 (41.7%)	0.37
Fetal_treatment	6 (40%)	5 (41.7%)	0.62	0.03	2 (50%)	0.57	3 (37.5%)	0.63
Fetal_treatment_days	0 (0–6.5)	0 (0–17.25)	0.51	0.23	9 (0–18.5)	0.35	0 (0–8.75)	0.63
Basket_catheter	4 (26.7%)	4 (33.3%)	0.52	0.04	2 (50%)	0.37	5 (25%)	0.67
Fetal_thoracocentesis	5(33.3%)	4(33.3%)	0.66	0.03	1(25%)	0.63	3(37.5%)	0.6

GW, gestational week; UA_pH, umbilical arterial pH; Fetal_CC_Days, the days from the onset of CC till birth; CC_start_week, the gestational age at the onset of CC.

### 3.3 Comparison between the ALIVE and the early-DEAD or the late-DEAD groups

Among the 12 patients who died, 4 died within 7 DOL (Early-DEAD), while the remaining 8 died later (Late-DEAD) (Table 1).

The rate of chromosomal abnormalities was significantly lower in the ALIVE group compared with both the Early-DEAD group (0% vs. 50%, respectively) and the Late-DEAD group (0% vs. 37.5%, respectively). Fetal_CC_Days was significantly shorter in the ALIVE group than in the Early-DEAD group (median 12 days vs. 48 days, respectively). In contrast, it was not significantly different between the ALIVE and the DEAD groups or between the ALIVE and the Late-DEAD groups. Moreover, the rate of fetal ascites was significantly lower in the ALIVE group than in the Late-DEAD group (20% vs. 87.5%, respectively).

### 3.4 Predictors of death

For the multivariate analysis, we selected potential predictors based on two criteria: 1) variables reported as risk factors in previous studies (male sex, GW, Apgar_5), and 2) variables that showed a statistically significant difference in our univariate comparisons between the ALIVE group and either the DEAD, Early-DEAD, or Late-DEAD groups (Table 1). The variables selected based on the latter criterion were chromosomal abnormality, fetal ascites, and Fetal_CC_Days.

The results of the univariate and multivariate logistic regression analyses are shown in Table 2. In the univariate analysis, chromosomal abnormality and fetal ascites were significantly associated with mortality. In the multivariate analysis using Firth's regression, only fetal ascites remained a statistically significant independent predictor of death (OR 1.69*10^1^, 95% CI 2.3–3.11*10^2^, *p* < 0.01).

**Table 2:** The predictors for death of CC.

	Univariate analysis			*p*	Multivariate analysis			*p*
	Odds ratio	2.5%ile	97.5%ile		Odds ratio	2.5%ile	97.5%ile	
Male	1.41	0.23	9.01	0.71	0.74	0.05	7.56	0.79
GW	0.77	0.46	1.14	0.2	0.69	0.31	1.12	0.13
Fetal_CC_Days	1	0.98	1.03	0.81	1	0.12	1.07	0.57
Chromosomal_abnormality	Inf	1.44	Inf	<0.01	5.3	0.3	9.23*10^2	0.28
Ascites	17.12	2.15	2.46*10^2^	<0.01	1.69*10	2.3	3.11*10^2^	<0.01
Apgar_5	0.84	0.55	1.23	0.37	0.99	0.46	2.51	0.96

Although chromosomal abnormality was not an independent predictor in the multivariate model, it was a strong predictor in the univariate analysis and is a clinically critical condition. Therefore, we constructed a predictive model for mortality using both chromosomal abnormality and fetal ascites. The performance of this model is detailed in the confusion matrix in Table 3. The model demonstrated a high prediction accuracy of 0.85, with a positive predictive value of 0.79, a sensitivity of 0.92, a negative predictive value of 0.92, and a specificity of 0.80.

**Table 3:** The confusion matrix of the prediction for death of CC.

		Prediction			
		Death	Alive		
Real data	Death	11	1		
	Alive	3	12		
				Accuracy	0.85
				positive predictive value	0.79
				Sensitivity	0.92
				Negative predictive value	0.92
				Specificity	0.8

### 3.5 Causes of death and pathological findings

Twelve patients died during hospitalization, and six (50%) of them underwent autopsy. Among these, one underwent only a lung necropsy, while the others underwent whole-body autopsy.

For all the Early-DEAD patients, the clinical cause of death was diagnosed as lung hypoplasia. In contrast, the causes of death among the eight Late-DEAD patients were more varied, including pulmonary hemorrhage (*n* = 2), cardiac failure or tamponade (*n* = 2), infection (*n* = 2), multiple organ failure (*n* = 1), and hypoxemia (*n* = 1) (Table 4). In six of these eight Late-DEAD patients, chylothorax had resolved before death.

**Table 4:** The details of dead cases.

		GW	Birth weight	CC_stop	DOL_at_CC_stop	DOL at death	Chromosomal examination	The other underlying condition	Clinical cause of death	Pathological findings
Early-DEAD	1	34	2,770	0		0	na	Family hisory: Myotonic dystrophy(mother)	Pulmonary hypoplasia	na
	2	31	2,458	0		4	na		Pulmonary hypoplasia, Pulmonary hypertension	Lymphagiectasia
	3	34	1,689	0	1	1	47XY +21		Pulmonary hypoplasia	na
	4	32	2,908	0	0	0	mosaicism with a supernumerary marker chromosome (mos 47,XX,+mar/46,XX)		Pulmonary hypoplasia	Pulmonary lymphagiectasia
Late-DEAD	1	29	1,486	0		61	47XY +21		Cardiac tamponade	na
	2	33	2,888	1	18	59	46XY	Noonan syndrome	Pulmonary hemorrhage	Lymphagiectasia
	3	33	1,738	1	31	88	47XY +21		Fungus infection	na
	4	36	3,611	1	13	379	na		Cardiac failure	na
	5	33	1,366	1	10	12	46XX		Hypoxemia	na
	6	35	1,660	1	3	388	46XYdel(7)(p13p15.1)	Pulmonary hemorrhage	Lymphagiectasia	
	7	32	1,478	1	12	60	na		Multiple organ failure	Lymphagiectasia
	8	33	2,874	0		159	na	Congenital multiple intestinal perforation	Liver failure, RS virus infection	Lymphagiectasia

GW, gestational week; CC_stop, resolution of chylothorax (1 = yes, 0 = no); DOL_at_CC_stop, days of life at resolution of chylothorax; DOL at death, days of life at death.

**Figure 1:**
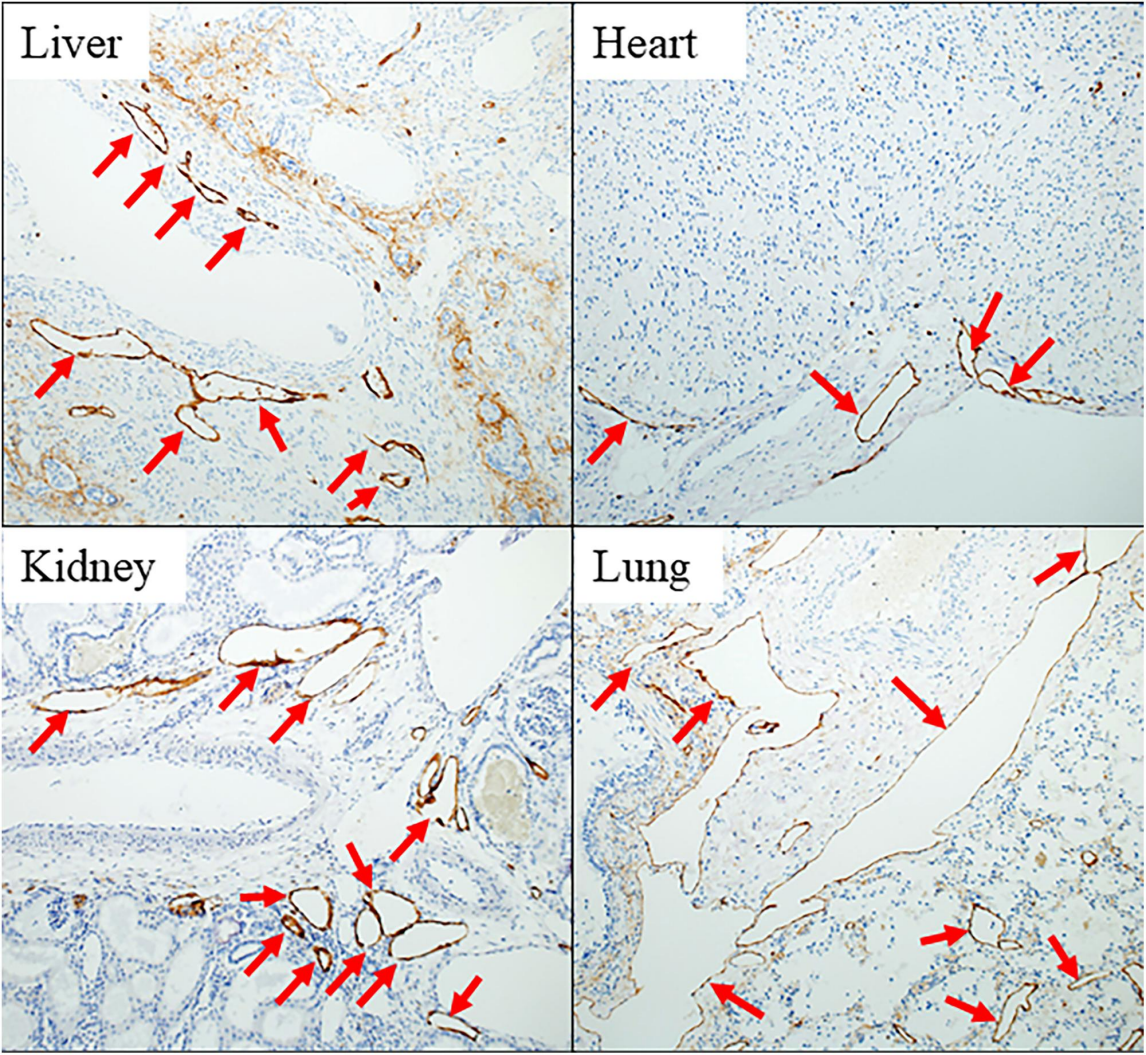
Pathological findings in case 2 in the late-DEAD group. Immunostaining for D2-40 of vital organs of the body stains the endothelium of lymphatic vessels brown. All red arrows indicate lymphatic ducts significantly dilated in each tissue.

Pathological examination revealed significant findings. All five whole-body autopsies showed systemic lymphangiectasia, and the lung necropsy showed pulmonary lymphangiectasia. The D2–40 re-stained samples from various organs, including the liver, heart, kidney, and lungs, all showed markedly dilated lymphatic vessels in the parenchyma (Figure 1).

## 4 Discussion

Although CC is a major cause of neonatal pleural effusion, it remains one of the most enigmatic diseases in neonatology because of its rarity. Recently, lymph-specific studies, such as lymphatic imaging (18–21) and pathological examination using D2-40 stains (15–17), have gradually advanced our understanding of lymphatic diseases, including CC. However, CC is still a critical condition. A systematic review reported a mortality rate of 28% among patients with CC (22). The present study aimed to clarify the pathogenesis of severe cases of CC by investigating the causes of death and risk factors in detail, and to obtain clues for the early detection and prevention of severe cases.

In this single-center retrospective study, we analyzed 27 cases of CC diagnosed based on strict hematological criteria of the pleural fluid (1, 2, 5, 7, 9–11). The mortality rate in our cohort was 44% (12 of 27 infants), which is comparable to the higher rates reported in previous multicenter studies and systematic reviews (1–11, 22). To our knowledge, this represents one of the largest case series to include detailed pathological findings from autopsies, providing a unique opportunity to investigate the pathophysiology of fatal CC cases.

When interpreting our findings, several limitations must be considered. First, chromosomal testing was not performed in all cases; thus, the true incidence of genetic abnormalities may be underestimated. Second, some patients had other severe underlying conditions, such as multiple intestinal perforations or clinically diagnosed Noonan syndrome, which may have acted as confounders and contributed to their deaths independently of CC. In this study, however, we evaluated only objectively confirmed chromosomal abnormalities as risk factors to maintain analytical consistency.

Consistent with previous studies, our univariate analysis identified several factors associated with mortality. These included chromosomal abnormalities and fetal ascites, which were linked to overall mortality, and a longer duration of hydrothorax *in utero* (Fetal_CC_Days), which was specifically associated with early neonatal death. However, some previously reported risk factors, such as fetal hydrops, preterm delivery, and low Apgar scores (4, 5, 8, 9), were not found to be significantly associated with mortality in our cohort, which may be attributable to our small sample size. A novel finding of our study is that the association with mortality was not limited to trisomy 21 (23) but extended to a broader range of chromosomal abnormalities.

To explore these associations further, we performed a multivariate analysis using Firth's logistic regression. This analysis revealed that fetal ascites was the sole independent predictor of mortality. The significant effect of chromosomal abnormalities observed in the univariate analysis was no longer statistically significant after adjusting for fetal ascites and other variables. This statistical finding suggests a clinically important relationship: rather than being a simple confounder, fetal ascites may act as a mediator in the causal pathway. That is, chromosomal abnormalities may lead to a poor prognosis by causing severe systemic conditions, such as Generalized Lymphatic Dysplasia (GLD), with fetal ascites serving as a critical indicator of this underlying pathology.

While our multivariate model identified fetal ascites as the sole independent predictor, the clinical utility of a strong univariate predictor like chromosomal abnormality for risk stratification should not be overlooked. Therefore, we constructed a predictive model for mortality using both chromosomal abnormality and fetal ascites. The performance of this model, detailed in the confusion matrix (Table 3), was high, with an accuracy of 0.85 and a particularly strong negative predictive value (NPV) of 0.92. The high NPV is clinically valuable, as it suggests that infants predicted to survive by this model have a high probability of doing so, which can inform parental counseling. To our knowledge, no previous studies have quantitatively evaluated the prediction accuracy for CC mortality using a confusion matrix. Our findings suggest that despite the result of the multivariate analysis, combining information on chromosomal abnormalities and fetal ascites provides a practical and reliable tool for identifying high-risk infants in a clinical setting.

Our findings suggest distinct mechanisms for early and late mortality. Early neonatal death was uniformly attributed to pulmonary hypoplasia. This is consistent with our risk factor analysis, which showed that a longer duration of hydrothorax *in utero* was specifically associated with early, but not late, mortality, likely due to prolonged compression of the developing lungs.

In stark contrast, the causes of death in the Late-DEAD group were varied, and critically, chylothorax itself had resolved in most of these infants before death. This crucial observation implies that in severe cases, CC is not merely an isolated thoracic duct problem but rather a symptom of a more profound systemic disease. Our pathological findings provide strong support for this hypothesis: systemic lymphangiectasia was identified in all autopsied cases. This suggests that an underlying condition, such as GLD, is the ultimate driver of mortality, leading to a gradual decline and death from various causes even after the initial chylothorax is managed.

This universal finding of lymphangiectasia in our fatal cases is critical. Lymphangiectasia is a known fatal condition, first described by Noonan in 1970 (24), characterized by the dilation of lymphatic vessels and leading to severe edema (25–28). While its pathogenesis is complex, it is increasingly considered to be a key manifestation of GLD (26). GLD is a systemic disorder that can cause a wide spectrum of lymphatic abnormalities, including not only CC and lymphangiectasia but also lymphangiomatosis, chylous ascites, and lymphedema (18, 19, 29–32).

This concept of GLD provides a compelling explanatory framework for our findings. The strong association we identified between fetal ascites and mortality may be best understood not as a direct consequence of CC, but as an indicator of underlying GLD. Furthermore, the fact that chromosomal abnormalities were also strongly associated with a poor prognosis suggests that such genetic conditions may predispose infants to developing systemic lymphatic dysplasia. This hypothesis is supported by previous reports describing GLD in association with conditions such as Noonan syndrome (30) and Down syndrome (33). As detailed evaluations of lymphatic flow continue to deepen our understanding of this pathophysiology (34), further research is warranted to elucidate the precise relationship between genetic abnormalities, GLD, and clinical outcomes in CC.

Our study has several strengths. By analyzing mortality based on its timing, we identified that a longer duration of *in utero* hydrothorax was a specific risk factor for early neonatal death, likely due to pulmonary hypoplasia. Furthermore, to our knowledge, this is one of the first studies to systematically investigate the causes of death in CC by incorporating detailed autopsy findings, including D2–40 staining, which revealed universal systemic lymphangiectasia in fatal cases. The primary strength, however, lies in integrating these pathological findings with a robust multivariate analysis. This approach allowed us to propose a potential pathophysiological model: our analysis identified fetal ascites as the strongest independent predictor, suggesting it may serve as a critical clinical indicator for a more severe underlying condition. This statistical finding provides a plausible link between a genetic predisposition (a wide range of chromosomal abnormalities) and the severe phenotype observed. We therefore hypothesize that a critical driver of mortality in these cases is an underlying GLD, for which fetal ascites acts as a key marker. This finding moves beyond a simple list of risk factors and offers a deeper hypothesis-generating insight into the mechanism of fatal CC. This suggests a refined clinical perspective: infants with CC accompanied by fetal ascites or chromosomal abnormalities might be managed not just for an isolated thoracic issue, but for a potential systemic lymphatic disorder, warranting intensive monitoring and comprehensive parental counseling.

However, our study also had several limitations. First, the retrospective, single-center design and small sample size limit the generalizability of our findings. The small sample size, while addressed in the multivariate analysis using Firth's regression, means that it must be acknowledged that the lack of statistical significance for some items may be owing to insufficient power. Furthermore, the associations reported in this observational study cannot establish definitive causation.

Second, as noted by the reviewers, using the same patient cohort for both model development and validation likely led to an overestimation of the predictive model's accuracy due to overfitting. The predictive model is therefore presented as hypothesis-generating, and its performance must be validated in an independent, multi-institutional cohort.

Third, autopsies were performed on only 50% of the deceased infants. Therefore, we could not confirm whether pathological lymphangiectasia and GLD were universally present in all patients with fatal CC. Furthermore, without pathological examinations on survivors, we cannot definitively conclude that lymphangiectasia occurred exclusively in fatal cases.

Fourth, several diagnostic and clinical variables were incomplete. As discussed, advanced lymphatic imaging (18, 19), which is useful for diagnosing GLD, was not performed, making it difficult to pre-mortem diagnose GLD or distinguish it from other lymphatic anomalies. Similarly, chromosomal testing was not performed in all cases, and we did not verify whether fetal ascites was chylous, both of which are important factors in our proposed model. Finally, the heterogeneity of treatments made it difficult to assess their impact on prognosis.

## 5 Conclusions

This study investigated the risk factors and pathological background of mortality in infants with CC. Our analysis identified fetal ascites as the strongest independent predictor of mortality, while a longer duration of hydrothorax *in utero* was a specific risk factor for early neonatal death. Pathological examination of fatal cases universally revealed systemic lymphangiectasia. We therefore hypothesize that a poor prognosis in CC is often driven by an underlying GLD, for which fetal ascites serves as a key clinical marker. This underlying systemic condition may be associated with genetic predispositions, such as chromosomal abnormalities. Hence, the presence of fetal ascites or chromosomal abnormalities should alert clinicians to the possibility of a severe, systemic lymphatic disorder, allowing for more intensive management and informed counseling. Future studies combining pathological, imaging, and genetic evaluations of CC are warranted to further elucidate its complex pathogenesis.

## Data Availability

The original contributions presented in the study are included in the article/Supplementary Material, further inquiries can be directed to the corresponding author.
